# Evaluation of Biochip System in Determining Isoniazid and Rifampicin Resistances of Mycobacterium Tuberculosis in Sputum Samples

**DOI:** 10.1371/journal.pone.0052953

**Published:** 2012-12-28

**Authors:** Wei Lu, Cheng Chen, Yan Shao, Jinyan Shi, Chongqiao Zhong, Dandan Yang, Honghuan Song, Guoli Li, Xiaoyan Ding, Hong Peng, Linyang Zhu, Yang Zhou, Limei Zhu

**Affiliations:** 1 Department of Chronic Communicable Disease, Center for Disease Control and Prevention of Jiangsu Province, Nanjing, Jiangsu Province, People’s Republic of China; 2 Department of Chronic Communicable Disease, Center for Disease Control and Prevention of Lianyungang, Lianyungang, Jiangsu Province, People’s Republic of China; 3 Laboratory of Tuberculosis, the Fourth People's Hospital of Lianyungang City, Lianyungang, Jiangsu Province, People’s Republic of China; Fundacion Huesped, Argentina

## Abstract

**Objective:**

To evaluate a biochip system in determining isoniazid and rifampicin resistances of *Mycobacterium tuberculosis* in sputum samples in a Chinese population.

**Methods:**

We assembled 907 sputum smeared positive specimens of tuberculosis patients in total. Each sample would be separated into two parts for culture and biochip assay simultaneously. And those cultured positive and having full drug resistance results would be used as reference. The McNemar χ^2^ test was adopted for evaluating the paired 2×2 table.

**Results:**

Compared with drug sensitivity test, the agreement rates of the two methods in detecting rifampicin and isoniazid resistances were 93.37% and 94.49%, respectively. The sensitivity and specificity of biochip in detecting isoniazid were 74.31% and 96.92%, respectively. Meanwhile, the sensitivity and specificity for rifampicin were 79.76% and 96.53%, respectively. For multi-drug resistance, the sensitivity and specificity were 64.62% and 97.75%, respectively.

**Conclusions:**

The biochip system is a rapid and accurate method for drug resistant tuberculosis diagnosis using sputum samples directly, especially for rifampicin resistance detection.

## Introduction

Tuberculosis (TB) as an infectious disease causes millions of death every year in the world, and it remains a major public health burden in developing countries [1], [2]. Meanwhile, China holds the second largest number of TB cases in the world and it was estimated that around 1 million new incident TB cases were emerging each year [2]. Recent years, it was in dilemma for TB treatment due to the increasing emergence of drug resistance of *Mycobacterium tuberculosis* (MTB) complex, which resulted in longer treatment duration but poor prognosis [3]–[6]. Among drug resistant (DR) TB, multidrug resistance (MDR), defined as resistant to at least isoniazid (INH) and rifampicin (RMP), is considered as one of the thorniest types [5]. However, in most countries, there was only less than 5% of the new and previously treated TB patients tested for MDR, and the number of MDR cases found only accounted for 16% of the total MDR TB estimated in 2010 [2]. According to Chinese national drug resistance surveillance of MTB complex in 2008, 5.7% in new case and 25.6% in retreated case were MDR TB, which indicated a serious epidemic of drug resistance [7]. Furthermore, diagnosis of MDR TB in China mainly depends on the conventional drug susceptibility test (DST), a complex and fragile method, usually needs several weeks to complete from a primary specimen and requires proficient technicians to interpret the results [8], [9].

With the insight of the molecular mechanisms of resistance to RMP and INH [10], [11], fast molecular detection of MDR TB became available based on the polymerase chain reaction (PCR) or hybridization technology [12]–[15]. Y. Guo et al. designed a biochip system for MDR detection based on the most common mutations in *rpo*B and *kat*G genes and the promoter region of the *inh*A gene [16]. In our study, we evaluated the efficacy of this biochip system in field work of MDR TB detection in China.

## Materials and Methods

### Samples Collection

Four counties and one downtown area of Lianyungang city, Jiangsu Province were selected to undertake case finding. We consecutively assembled all smear-positive TB patients by Ziehl-Neelsen sputum smear method from January, 2011 to April, 2012. Finally, 907 smear positive cases were enrolled in this study, including 666 new incident cases and 241 retreated cases. This study was approved by the ethics committee of Center for Disease Control and Prevention of Jiangsu Province, and all participants provided their written informed consents before enrollment.

### Sputum Culture and DNA Extraction of *Mycobacterium tuberculosis*

The sputum samples were decontaminated with an equal volume of NaOH-NALC (including 4% NaOH, 2.94% Sodium Citrate and 0.5% (w/v) NACL) and mixed for 1–5 minutes by vortex before incubation for 15 minutes at room temperature. Add 1 ml liquefied sputum sample to a 1.5 ml centrifuge tube for DNA extraction and the surplus was cultured on Lowenstein-Jensen (LJ) culture media. Lowenstein Jensen (LJ) culture media were incubated at 37°C and observed on the 3rd day to detect contamination. Subsequently, we recorded the growth on LJ media each week until eighth week.

The DNA extraction of all sputum examples followed the manufacturer’s protocol of CapitalBio Universal Kit (CapitalBio, Beijing, China) as previously reported [16].

The 1.5 ml centrifuge tubes with liquefied sputum samples were centrifuged at 12,000×g for 5 minutes and then washed by 0.9% (w/v) saline. The pellet was mixed with the DNA extraction reagent (CapitalBio) and treated by the Extractor™ 36 (CapitalBio) at maximum speed for 5 minutes. The extraction tube was incubated at 95°C for 5 minutes before a brief centrifugation. Finally the total DNA was stored at −20°C until use.

### Biochip MDR Assay

The full set of biochip system includes a biochip, apparatus for sample preparation, chip hybridization, washing and data acquisition, and dedicated software for automated diagnosis [16]. Determination of MDR of MTB complex using biochip test was undertaken according to the manufacturer’s instructions (CaptialBio, Beijing, China). Multiplex asymmetric polymerase chain reaction (MAPCR) was performed in a TC-96/G/H(b) thermal cycler. The PCR products were hybridized with a biochip in a BioMixer II three dimensional tilting agitator and a hybridization oven. After wash and spin by an automated Slide Washer-8 (CapitalBio), the biochip slides were analyzed with LuxScan-10K confocal laser scanner and *Mycobacterium tuberculosis* drug resistance detection array test system software, and the drug resistance pattern of INH and RMP can be referred to the previous report [16]. Ahead of the launch of the study, all technicians were trained by the National TB reference laboratory and confirmed by proficiency test. All the biochip results were compared with conventional DST results.

### Drug Susceptibility Test

The drug susceptibility test (DST) was performed according to the proportion method as recommended by WHO/IUATLD [17]. The concentrations of anti-tuberculosis drugs were 0.2 µg/ml for INH and 40 µg/ml for RMP. As a parallel test, the p-nitrobenzoic acid (PNB) was utilized for *non-tuberculosis Mycobacterium* (NTM) identification. Growth in LJ medium containing PNB indicates that the bacilli do not belong to the *Mycobacterium tuberculosis* complex [18]. For internal and external quality controls, a standard H37Rv strain was introduced for each batch of culture and the proficiency of DST was supervised by National TB reference laboratory of China. The proficiency rate of DST for INH and RMP was 93.33% and 93.33% respectively, and the reproducibility was 100% for both drugs.

### Statistical Analyses

The McNemar χ^2^ test was adopted for evaluating the paired 2×2 table. Meanwhile, we calculated the agreement of biochip assay compared with DST, and all of the statistics were performed by SPSS 17.0 software (Statistical Package for the Social Sciences Inc, Chicago, IL, USA).

## Results

### Screen the Specimens by Culture and DST

**Figure 1:**
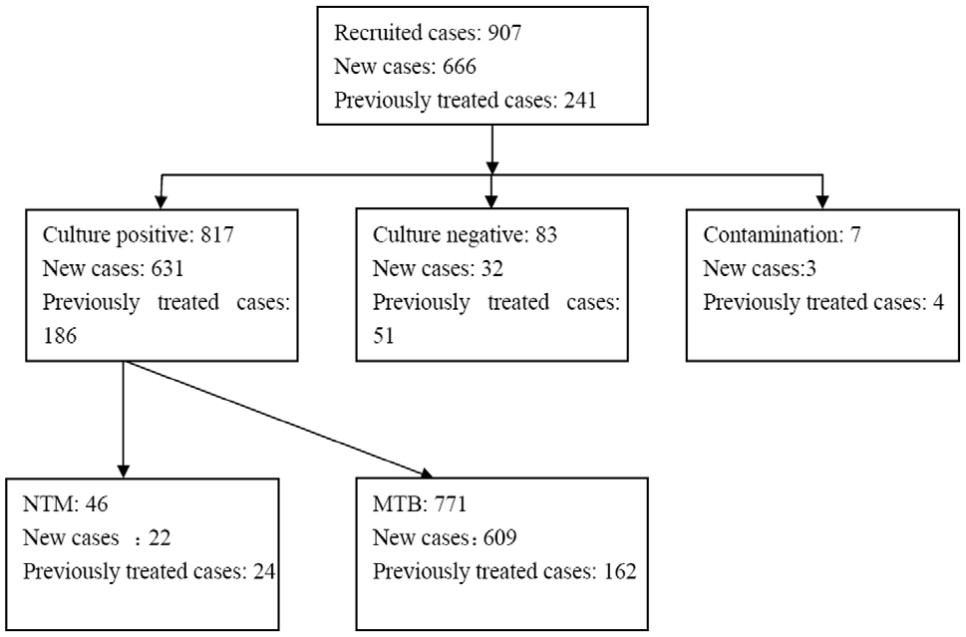
Flow chart of Mycobacterium tuberculosis complex screening.

Among 907 sputum smear positive TB patients recruited in this study, 817 (90.08%) cases were culture-positive, 83(9.15%) cases were culture-negative and 7(0.77%) cases were contaminated. Thus, 817 cultured positive samples were running into DST and NTM identification. According to PNB test, we found 46 cases were confirmed as NTM. Finally, 771 samples reported DST results (Figure 1).

### Results of Biochip System Assay in Clinical Sputum

All of 907 sputum samples were tested by the biochip system to detect RMP and INH resistance. Because of the confirmed 771 DST results, we only included the corresponding 771 results of biochip system.

Among 771 sputum samples, 690 biochip results for RMP detection was successful (Table 1), and the rest 81 was failed. Thus, 690 samples with both full results were taken for analysis. The agreement rate was 94.49% for DST and biochip method for validated RMP resistance detection. The sensitivity and specificity for biochip system in detecting RMP resistance pattern were 79.76% and 96.53%, compared with DST results. McNemar χ^2^ test showed that the two tests demonstrated no difference (*P* = 0.5164, Kappa = 0.7476).

**Table 1:** Comparison of DST and biochip for rifampicin resistance pattern*.

	DST assays for RMP		
	Susceptible	Resistant	
Biochip test			Agreement rate: 94.49%
Wild type	585	17	602
Mutant type	21	67	88
total	606	84	690

* DST: drug susceptibility test; RMP: rifampicin. McNemar χ^2^ test, *P* = 0.5164, Kappa = 0.7476.

For INH resistance pattern detection (Table 2), 694 samples gave validated results of biochip, and the rest 77 sputum samples were failed. The sensitivity and specificity for biochip system in detecting INH resistance were 74.31% and 96.92%, respectively, compared with DST results, and the agreement rate was 93.37% for validated results. McNemar χ^2^ test showed that the two tests demonstrated no difference (*P* = 0.1404, Kappa = 0.7400).

**Table 2:** Comparison of DST and biochip for isoniazid resistance pattern*.

	DST assays for INH		
	Susceptible	Resistant	
Biochip test			Agreement rate: 93.37%
Wild type	567	28	595
Mutant type	18	81	99
total	585	109	694

* DST: drug susceptibility test; INH: isoniazid. McNemar χ^2^ test, *P* = 0.1404, Kappa = 0.7400.

Finally, 687 samples with successful biochip results of INH and RMP were qualified for MDR calculation (Table 3). And we found the sensitivity and specificity was 64.62% and 97.75%, respectively, compared with DST results. Meanwhile, Mcnemar χ^2^ test demonstrated that the two test showed no difference (*P* = 0.1390, Kappa = 0.6649). The agreement rate of the two methods with validated results was 94.61%.

**Table 3:** Comparison of DST and biochip for MDR*.

	DST assays for MDR		
	none-MDR	MDR	
Biochip test			Agreement rate: 94.61%
none-MDR	608	23	631
MDR	14	42	56
total	622	65	687

* MDR: Multidrug resistance, defined as resistant to at least isoniazid and rifampicin. McNemar χ^2^ test, *P* = 0.1390, Kappa = 0.6649.

## Discussion

In our study, we found the biochip system for MDR assay was in high concordance compared with DST. Thus, the biochip system might be a potential accurate and rapid method for MDR case finding in high TB burden areas.

It is well-known that MDR TB is one of the major obstacles to the global TB control. It is not because of the disease is untreatable, but because of long treatment duration and expensive regimen, complicated with severe adverse reaction after medication. China national survey of drug resistance of TB conducted in 2007 demonstrated that 5.7% of new incident TB and 25.6% of previously treated tuberculosis cases were MDR TB, respectively. Moreover, the situation of XDR (defined as resistant to at least isoniazid, rifampicin, ofloxacin, and kanamycin) was grim in China, as it was estimated that 8% of MDR-TB was XDR [7]. According to our survey of drug resistance of TB in 2008, there was a high prevalence of MDR TB in Jiangsu Province, and the MDR TB rate was 7.63% in new cases and 33.07% in previously treated patients. Thus, the high prevalence of MDR TB in Jiangsu Province hindered TB control and increased the demand of sanitation [6]. So an accurate and rapid method for MDR detection was in urgent need.

As all we known, susceptibility testing of MTB isolates by phenotypic methods is time-consuming, which usually takes two to three months [9]. However, molecular detection methods could obtain results in much shorter time. With reduced diagnosing time, MDR TB patients could be treated timely with proper regimen, and the transmission of drug resistant strains could be reduced as little as possible.

In this study, we evaluated a biochip system designed for rapid molecular determination of MDR TB using a panel of 907 sputum specimens. It usually took 6 hours in average for the whole process.

Meanwhile, our study revealed a high consistency between the biochip system and DST. The agreement rates were 94.49% and 93.37% respectively for RMP and INH resistance detection. McNemar test χ^2^ indicated that there was no significant difference on drug resistance assay between biochip system and DST.

Molecular diagnostic methods such as the Genotype MTBDRplus assay (Hain Lifescience GmbH, Nehren, Germany) has been evaluated for INH and RMP resistance several years ago [19], [20], and the concordance rate exceeded 90% compared with DST for clinical specimens. However, the results interpretation was mainly determined by technicians themselves, and the results maybe discrepant among operators. Previously, the instrumentation and microarray components had been independently evaluated for detection of other infectious entities, such as staphylococcal isolates [21]. Recently, the technology of microarray had been implemented in identification of MTB complex [22], [23], which gave rapid and accurate detection of MTB complex. Meanwhile, microarray had been used in drug resistance TB finding in near years by [14], [24]. In our study, we evaluated the biochip system in a large sample size in China, which gave the evidences and experiences for using this rapid method for MDR TB detection.

The biochip system has some apparent advantages in drug resistance detection. Firstly, the biochip contains probes for most of the frequent mutation types and may add new resistant mutation probes for improving the sensitivity of drug resistance. Secondly, the biochip system provides an automated determination of results which could avoid subjective judgment. Although the biochip system was performed well in this study, we still found some limitations in the process of evaluating the method. First, during the screening period of this study, we found the ratio of culture-negative (83/907 = 9.15%) was a little higher than common situation although acceptable. However, further analysis revealed that 61.4% culture-negative cases were previously treated patients, and we postulate inactive mycobacterium bacillus from retreated patients may induce negative culture results. Secondly, we only evaluated the smear positive patients; those smeared negative TB patients were not evaluated in this study, and those smeared negative but cultured positive subjects need to be evaluated for this biochip system. Also, apart from INH and RMP susceptibility assay, culture method is needed for other drugs susceptibility testing, such as ethambutol and streptomycin.

### Conclusion

Multidrug resistance aggravates the spread of tuberculosis especially in heavy-burden countries and impedes the progress of global TB control strategy. Therefore, the expanded capacity to rapid and accurate detection of MDR TB is a priority for TB control. The biochip system designed for determination of MDR TB is appropriate to achieve the above goal. With the satisfactory concordance rate and fast procedure within 6 hours, the biochip system will compress the diagnosis course distinctly and make MDR TB patients to get efficient chemotherapy much earlier.
